# Enzymes that catalyze cyclization of β-1,2-glucans

**DOI:** 10.1007/s00253-025-13429-x

**Published:** 2025-02-20

**Authors:** Masahiro Nakajima, Sei Motouchi, Nobukiyo Tanaka, Tomoko Masaike

**Affiliations:** Department of Applied Biological Science, Faculty of Science and Technology, Tokyo University of Science, 2641 Yamazaki, Noda, Chiba 278-8510 Japan

**Keywords:** Cyclic β-1,2-glucan synthase, β-1,2-Glucan, Osmoregulated periplasmic glucans, Glycoside hydrolase family, Transglycosylation

## Abstract

**Abstract:**

β-1,2-Glucans are physiologically important polymers for interactions such as symbiosis and pathogenesis between organisms and adaptation to environmental changes. However, rarity of β-1,2-glucans in nature limits exploration of related enzymes. Recently, many β-1,2-glucan-degrading enzymes have been found after identification of a novel phosphorylase acting on β-1,2-glucooligosaccharides. The expansion of the repertoire has reached revelation of the cyclization mechanism of cyclic β-1,2-glucan synthase and led to finding of new enzymes catalyzing cyclization of β-1,2-glucans in a manner different from cyclic β-1,2-glucan synthase. In this review, we mainly focus on newly found enzymes that catalyze cyclization of β-1,2-glucans along with existence of β-1,2-glucan-associated carbohydrates in nature and introduction of the repertoire of β-1,2-glucan-degrading enzymes.

**Key points:**

• *Newly found domain which cyclizes β-1,2-glucan created a new glycoside hydrolase family.*

• *Cyclization is performed with a unique mechanism.*

• *α-1,6-Cyclized β-1,2-glucan is produced by an enzyme in another newly found family.*

## Introduction

Carbohydrates are biopolymers important not only as energy sources but also as storage polymers, cell skeletons, and for interactions such as infection and immune response between organisms (Molina et al. 2024a, 2024b; Qu et al. 2025). Widely diverse physiological roles are assumed to be attributable to the complexity of carbohydrate structures given by a variety of component monosaccharides, isomers derived from various linkage positions of glycosidic bonds and orientations of anomers, and various kinds of their modification. Enzymes related to carbohydrates with various functions and structures have emerged extensively by molecular evolution. Thus, it makes sense that we have still been unable to elucidate minor but important carbohydrates sufficiently, let alone enzymes degrading or synthesizing them.

In terms of glucose polymers, a β-1,4-linked polymer is called cellulose, the most abundant component of biomass in nature. β-1,3-linked polymer is also commonly found in plants, bacteria, seaweeds, and fungi (Stone 2009). In addition, an α-1,4-linked polymer (with some α-1,6-linkages) is called starch, which is one of the major storage polysaccharides and core industrial carbohydrates (Cosgrove et al. 2024; Ahmad et al. 2024). Meanwhile, β-1,2-glucans are compounds first found in *Rhizobium radiobacter* (formerly *Agrobacterium tumefaciens*) in 1940 as an extracellular polysaccharide resulting in crown galls of plant roots (McIntire et al. 1940). A wide distribution of β-1,2-glucans in spite of their minute amounts in nature suggests their physiological importance. Although β-1,2-glucans were usually found in cyclic forms, enzymes responsible for cyclization of linear β-1,2-glucans and their molecular mechanisms have remained unknown until recently. In this review, we mainly feature enzymes cyclizing β-1,2-glucans while referring to β-1,2-glucans in nature and describing the background to exploration of β-1,2-glucan-degrading enzymes.

## β-1,2-Glucan-associated carbohydrates in nature

### Cyclic β-1,2-glucans

**Fig. 1:**
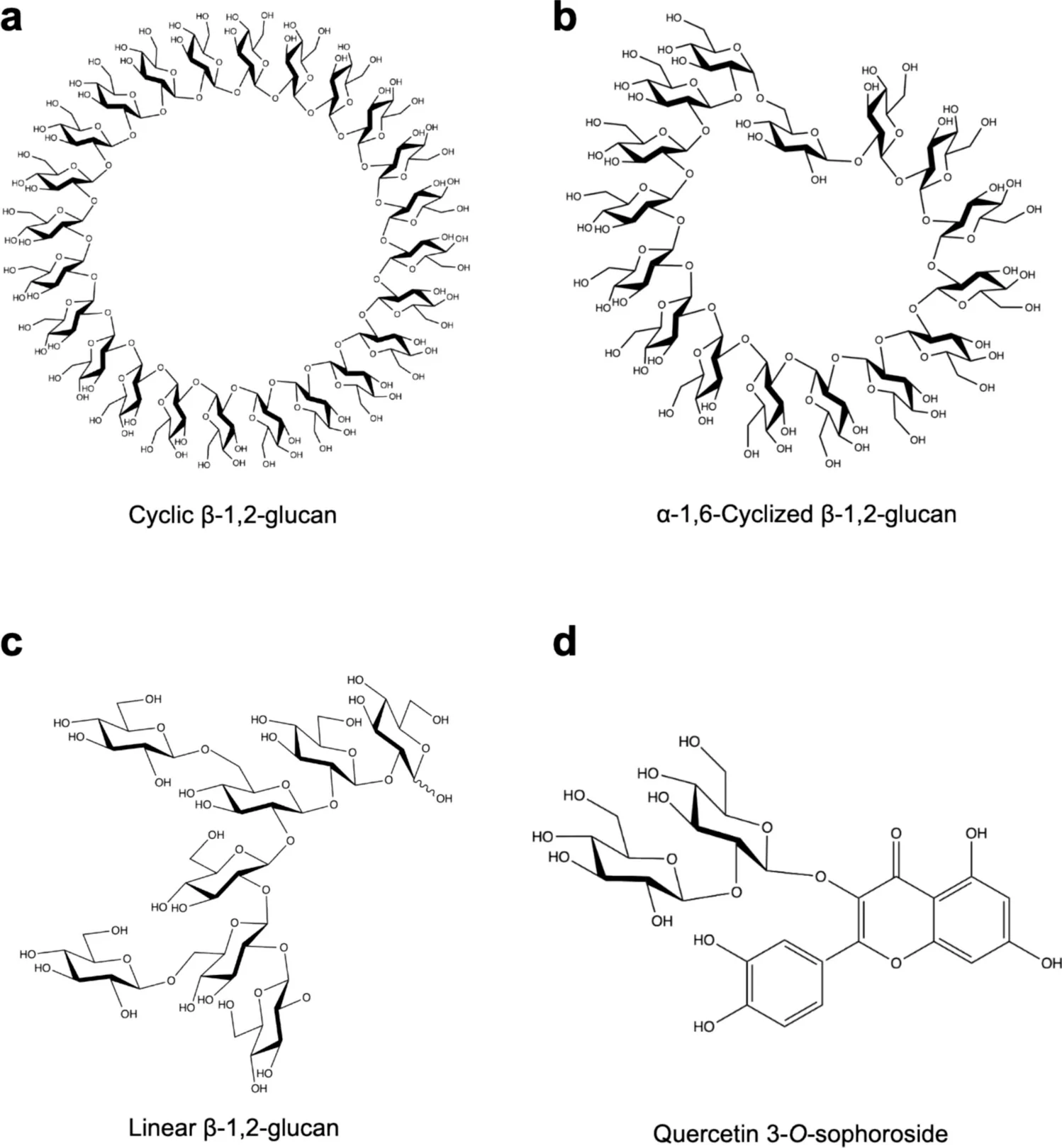
β-1,2-Glucans and β-1,2-glucooligosaccharides found in nature. **a** An example of cyclic β-1,2-glucan. **b** α-1,6-Cyclized β-1,2-glucan with DP16 found from *X. campestris*. **c** An example of a short linear β-1,2-glucan with side chains of β-1,6-glucose units. **d** Quercetin 3-*O*-sophoroside, an example of β-1,2-oligoglucoside

As described above, cyclic β-1,2-glucans (CβGs) are the first β-1,2-glucans ever found. Their degrees of polymerization (DPs) produced from Gram-negative bacteria such as *Agrobacterium*, *Brucella*, and *Sinorhizobium* are around 20 (Hisamatsu et al. 1982; Briones et al. 1997) (Fig. 1a), and modification with acids such as succinate is often found in CβGs (Roset et al. 2006; Guidolin et al. 2015). CβGs are also found in edible green microalgae such as *Chlorella pyrenoidosa* (Suárez et al. 2008). CβGs are known as symbiotic or pathogenetic factors and also as compounds responsible for osmoregulation and storage of iron. For example, after *B. abortus* invades host cells, the bacterium makes the host cell form a *Brucella*-containing vacuole. CβGs prevent the vacuole from fusing with a lysosome, which gives the bacterium resistance against phagocytosis by a macrophage (Arellano-Reynoso et al. 2005). CβGs are necessary for hypoosmotic adaptation in *Rhizobium meliloti* (Dylan et al. 1990). Moreover, *Sinorhizobium* sp. strain NGR234 adapts to hypoosmotic environments and achieves symbiosis with its host by accumulating CβGs in the periplasm (Gay-Fraret et al. 2012).

### α-1,6-Cyclized β-1,2-glucans

CβGs with one α-1,6-glucosidic linkage (α-1,6-cyclized β-1,2-glucans, CβGαs) are also found (Fig. 1b). Phytopathogens *Ralstonia solanacearum* and *Xanthomonas campestris*, and a photobacterium *Cereibacter* (formerly *Rhodobacter*) *sphaeroides*, all produce CβGαs with DPs of 13, 16, and 18, respectively (Bohin 1996; Wieruszeski et al. 2001; Talaga et al. 2002; Wanke et al. 2021). CβGαs are often modified with succinyl and/or acetyl groups as in the case of CβGs (Bontemps-Gallo et al. 2017). In *X. campestris*, CβGα plays a key role in virulence because a gene deletion mutant strain that cannot produce the CβGα loses pathogenicity in model experimental plants such as *Arabidopsis thaliana* and *Nicotiana benthamiana* (Rigano et al. 2007). In *X. campestris* pv. *campestris* 8004, deletion of *ndvB* gene which is related to production of CβGα resulted in growth defect under shortage in iron (Javvadi et al. 2018). Although other CβGαs are also found in the periplasm, their detailed physiological roles are unclear, and their functional differences dependent on the DPs remain uncertain (Talaga et al. 2002).

### Linear β-1,2-glucans

Osmoregulated periplasmic glucans (OPGs) in *E. coli* are linear β-1,2-glucans (LβGs) (Kennedy 1982). OPGs are also observed in *Salmonella enterica* serovar Typhimurium (Bhagwat et al. 2009), *Pseudomonas aeruginosa* (Mahasenan et al. 2020), and *Pseudomonas syringae* (Talaga et al. 1994). A characteristic feature of these LβGs is that they possess short main chains and β-1,6-linked branches of glucose units. The DPs of the main chain are between 6 and 13 (Fig. 1c) (Bontemps-Gallo et al. 2017). The OPGs are modified with succinate, phosphoglycerol, and phosphoethanolamine (Bontemps-Gallo et al. 2017). However, the modified positions in the OPG are not identified to our knowledge. Production of OPGs is quite critical for some microorganisms. OPGs from *Yersinia enterocolitica* contribute to antibiotic resistance (Meng et al. 2020). A silkworm is resistant to *E. coli* that is considered to lack OPGs due to the defect of a gene related to its production (Murakami et al. 2021). In a phytopathogen *Dickeya dadantii*, OPGs induce production of cell wall-degrading enzymes (Cochard et al. 2023). In *E. coli*, β-1,2-glucan chains are produced in the inner membrane and are released into the periplasmic space by OpgH (Bontemps-Gallo et al. 2017). Deletion of genes related to OPG synthesis including *opgH* in *E. coli* resulted in a remarkable increase in production of an exopolysaccharide, colanic acid (Wu et al. 2024). It is reported that OpgH is crucial for regulation of morphology in *Caulobacter* (Daitch et al. 2024). OpgG and OpgD, which are assumed to be located in the periplasm, are found to be involved in OPG synthesis. OpgG is indispensable for OPG synthesis because a *ΔopgG* strain loses ability to produce OPG (Bontemps-Gallo et al. 2017). A *ΔopgD* strain produces OPG with main chains longer than those of a wild-type strain. Adjustment of the chain lengths was suggested to be a physiological role of OpgD based on this observation (Lequette et al. 2004). However, biochemistry of OpgG and OpgD had not been unraveled until the authors revealed their enzymatic functions recently.

β-1,2-Glucooligosaccharides (Sop_n_s, n is DP; “Sop” is derived from sophorooligosaccharide, an alternative name of β-1,2-glucooligosaccharide)^1^ are also found as secreted oligosaccharides in *Acetobacter* (Amemura et al. 1985). Recently, there has been a report that Sop_3_ triggers pattern-triggered immunity in plants by increasing reactive oxygen species (Fuertes-Rabanal et al. 2024). However, the number of reports on the existence of Sop_n_s is much less than those of β-1,2-glucans.

### β-1,2-Oligoglucosides

Sop_n_s are mainly found in the structure of glycosides such as stevioside, quercetin 3-*O*-sophoroside, kaempferol-3-*O*-sophoroside, and ginsenoside, a kind of saponin (Zhang et al. 2022, 2025; Żurek et al. 2022; Chen et al. 2024) (Fig. 1d). Although mainly found compounds are sophorosides (a Sop_2_ modifying an aglycone), compounds with Sop_3_ are also found in Chinese cabbage (*Brassica rapa* L. subsp. *chinensis*) and Nightshade (*Solanum retroflexum* Dun) (Managa et al. 2020). Kaempferol-3-*O*-sophoroside and cultivated mountain ginseng show various medicinal properties such as anti-tumor, anti-oxidative activity, anti-allergic activity, anti-diabetic activity, and mitigation effect on acetaminophen-induced hepatotoxicity (Kim et al. 2012; Mohamed et al. 2024). Antibacterial activity is also reported in petal extract containing kaempferol-3-*O*-sophoroside from *Crocus sativus* L. (Naim et al. 2022). The molecular mechanism of the effects of this compound is also studied by in silico analyses (docking with potential target proteins) (Frusciante et al. 2024). Quercetin 3-*O*-sophoroside isolated from *Pisum sativum* shoots showed effects of protection against liver injury induced by chemicals (Wang et al. 2020).

## Expansion of the repertoire of β-1,2-glucan-degrading enzymes

To understand physiological roles of β-1,2-glucans in detail, it is essential to investigate biochemical functions and tertiary structures of enzymes which degrade or synthesize β-1,2-glucans. Although a synthetic mechanism of CβGs had been studied, mechanisms of cyclization of linear β-1,2-glucans were unknown until the studies were published in 2024 (Sedzicki et al. 2024; Tanaka et al. 2024). Furthermore, no gene encoding β-1,2-glucan-degrading enzymes had been identified.

Phosphorolysis of β-1,2-glucans by cyclic β-1,2-glucan synthase (CGS) from *Agrobacterium* had been reported to be a degradation process as described in the later section. Nevertheless, this activity was merely one of the steps in glucan synthesis. 1,2-β-Oligoglucan phosphorylase (SOGP) phosphorolyzing Sop_n_s with DP 3 or higher from *Listeria innocua*, a Gram-positive bacterium was discovered in 2014 (Nakajima et al. 2014). Because the reactions of glycoside phosphorylases are generally reversible, synthetic (reverse) reactions are used for oligosaccharide synthesis. Thus, methods of large-scale production of LβGs were developed by taking advantage of the reverse reaction of SOGP (Abe et al. 2015). This achievement of obtaining substrate LβGs opened new strategies to explore β-1,2-glucan-associated enzymes.

The genes encoding β-1,2-glucanase (SGL) were identified in *Chitinophaga pinensis*, a Gram-negative bacterium, and *Talaromyces funiculosus*, a filamentous fungus, using the LβGs synthesized by SOGP as a sole carbon source for growth of these microorganisms (Abe et al. 2017; Tanaka et al. 2019). The amino acid sequences of both enzymes were different from known glycoside hydrolases (GHs) (Drula et al. 2022). Therefore, the CpSGL and TfSGL homolog groups were announced to be classified into novel GH families (GH144 and GH162, respectively). With enzymes catalyzing synthesis and degradation of oligosaccharide in hand, we obtained means to prepare LβGs with controlled DPs (Nakajima et al. 2021). In addition, referring to our reports on large-scale production methods and the discovery of the SGLs, LβGs had become commercially available.

Identification of the *sgl* genes of GH144 and GH162 led to the expansion of the repertoire of β-1,2-glucan-associated enzymes. An exo-type β-1,2-glucan degrading enzyme releasing Sop_2_ from non-reducing end (EC 3.2.1.214) was found in GH144 homologs (Nakajima 2023). β-1,2-Glucosyltransferase (EC 2.4.1.391) and carbohydrate-binding subunit in ABC transporter which specifically binds Sop_n_s were found in *sgl* gene clusters (Kobayashi et al. 2022; Nakajima 2023). Recently, a cryo-EM structure of ABC transporter of CβGs was revealed (Sedzicki et al. 2022). A β-glucosidase which preferably degrades Sop_n_s was found in *L. innocua*, and then a β-glucosidase preferable for both Sop_n_s and LβGs was found in *Bacteroides thetaiotaomicron* (Nakajima 2023). The latter one and CpSGL are used for the analysis of activity of CGS (detection of CβGs production). These newly found enzymes have also been structurally solved and structure–function relationships were revealed (Nakajima et al. 2017; Nakajima 2023).

## Enzymes synthesizing β-1,2-glucan-associated carbohydrates

### Constitution of CGS

Genes encoding enzymes that synthesize CβG were cloned from *R. radiobacter*, *S. meliloti*, and *B. abortus* independently (Castro et al. 1996; Iannino et al. 1998). The identified genes were eventually found to be homologous to each other. Although a term *ndvB* is sometimes used as a name of the gene or an annotation, The term *cgs* is used in the present review. CGSs are large membrane proteins of approximately 300 kDa. Among them, the CGS from *B. abortus* is one of the most characterized CGSs. Mutational analyses of the CGS revealed the detailed mechanisms in the reaction steps (Ciocchini et al. 2007). The CGSs produce CβGs through 4 steps: initiation, elongation, adjustment, and cyclization. Initiation is a step in which a glucose binds to a CGS covalently. In the second step, elongation, a β-1,2-glucan chain is elongated by attaching donor substrate UDP-glucose units to the bound glucose unit. In the third step named adjustment, phosphorolysis of the elongated chain is performed to optimize the DP. In the final cyclization step, the linear chain is cyclized to complete the production of CGS. Once generated, the CβGs are no longer cleaved by the CGS.

A CGS consists of three regions: N-terminal, C-terminal, and middle regions. An N-terminal region contains a glycosyltransferase (GT) family 2 domain (Ciocchini et al. 2006). In the case of CGS from *R. radiobacter*, this region is further divided into two domains, N-terminal and GT2 domains (Sedzicki et al. 2024). The latter GT2 domain carries out the initiation and the successive elongation process. The C-terminal region is a GH94 domain that shows phosphorolytic activity. This domain performs adjustment, the third step in the CGS production. Contrarily, the other middle region had been functionally unknown, and how the cyclization of substrates occurs had also remained unknown until 2023.

Homologs of this domain, whose functions are unknown, form a large phylogenetic group. Analysis by PSI-BLAST in search for related groups revealed that this group forms a superfamily with GH144 and GH162, families of SGLs (Tanaka et al. 2024). Moreover, the general acid residue in TfSGL (GH162) is highly conserved among GH144 and the group with unknown functions. In contrast, the general base residue in TfSGL is not conserved among the other two groups. Considering the fact that only one of the catalytic residues is conserved, the group with unknown functions is expected to have some enzymatic function if not completely the same as SGLs. Recently, the corresponding unknown domain of CGS homolog from *Thermoanaerobacter italicus* (TiCGS), a Gram-negative bacterium, has been reported to have the activity of cyclizing linear β-1,2-glucans, which is described in detail in the next section (Tanaka et al. 2024).

### Function and structure of the conserved domain in the middle region of TiCGS (TiCGS_Tg_)

When the recombinant TiCGS_Tg_ (Tg represents transglycosylation) alone expressed in *E. coli* and purified was incubated with LβGs, β-glucosidase-resistant molecules were produced. TiCGS_Tg_ did not act on other polysaccharides, suggesting that the specific substrates of this enzyme are LβGs. ESI–MS analysis showed that the products have the same molecular weights as cyclic glucans with DP of 17–26. Moreover, ^1^H NMR showed the same pattern of the chemical shifts as those of reference CβGs. No anomeric proton of a glucose unit of α-anomer was detected. These results clearly evidenced that the products are CβGs with DP of 17–26, and TiCGS_Tg_ follows the anomer-retaining mechanism in the course of CβG production.

**Fig. 2:**
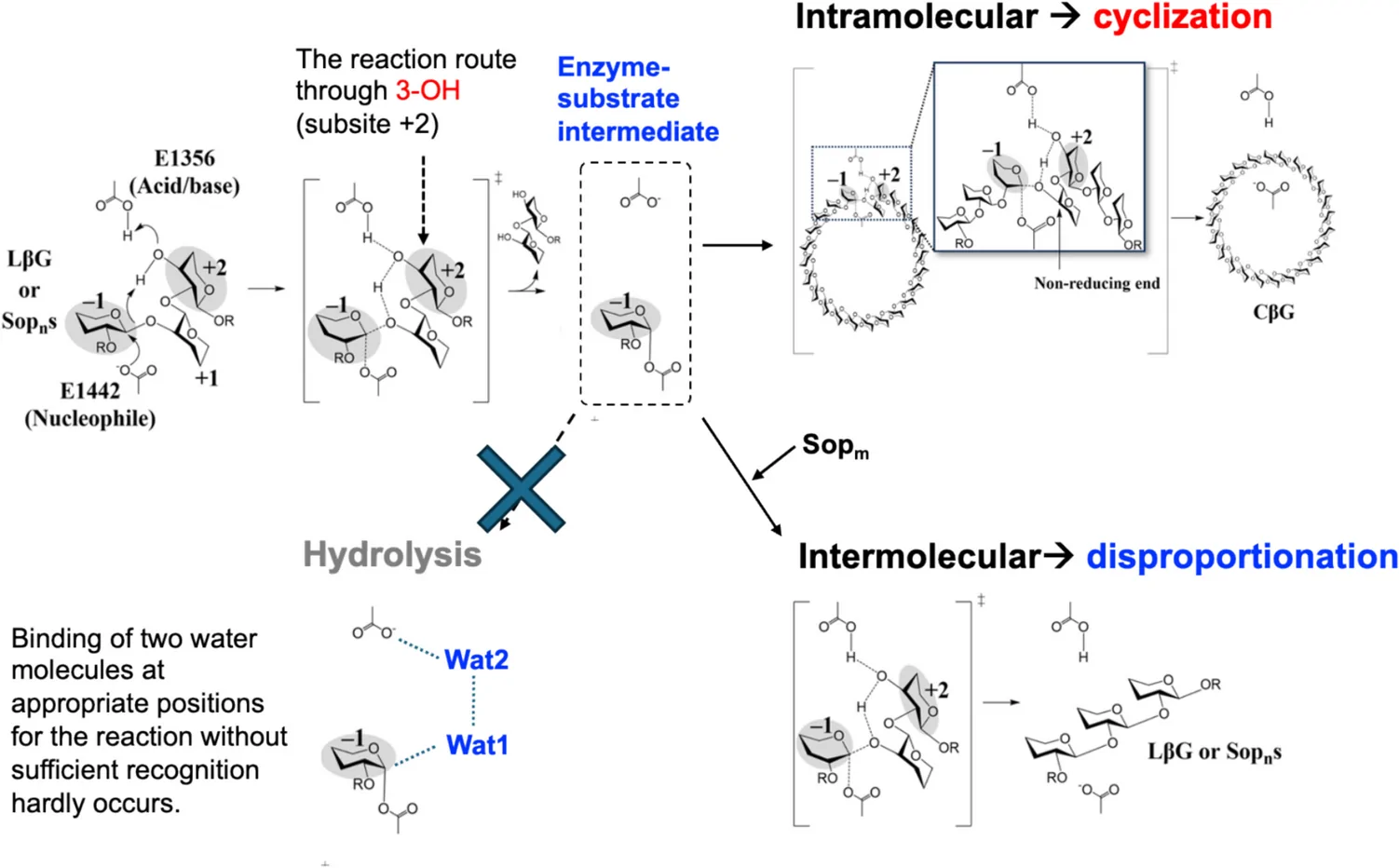
The reaction scheme of TiCGS_Tg_ explaining the elementary steps. TiCGS_Tg_ has unique elementary steps in the cyclization process; enzyme–substrate intermediate is formed by protonation to a scissile bond oxygen atom through 3-OH at subsite +2 by E1356 as an acid and nucleophilic attack of E1442 (nucleophile) to an anomeric carbon in the first step called glycosylation step. In the subsequent step called deglycosylation, cyclization or disproportionation occurs depending on whether nucleophilic attack to the enzyme–substrate intermediate occurs in intra- or inter-molecular manner. The nucleophile is activated by deprotonation through 3-OH at subsite +2 by E1356 as a base. Hydrolysis cannot occur presumably because either Wat1 or Wat2 is fixed insufficiently for the efficient reaction

**Fig. 3:**
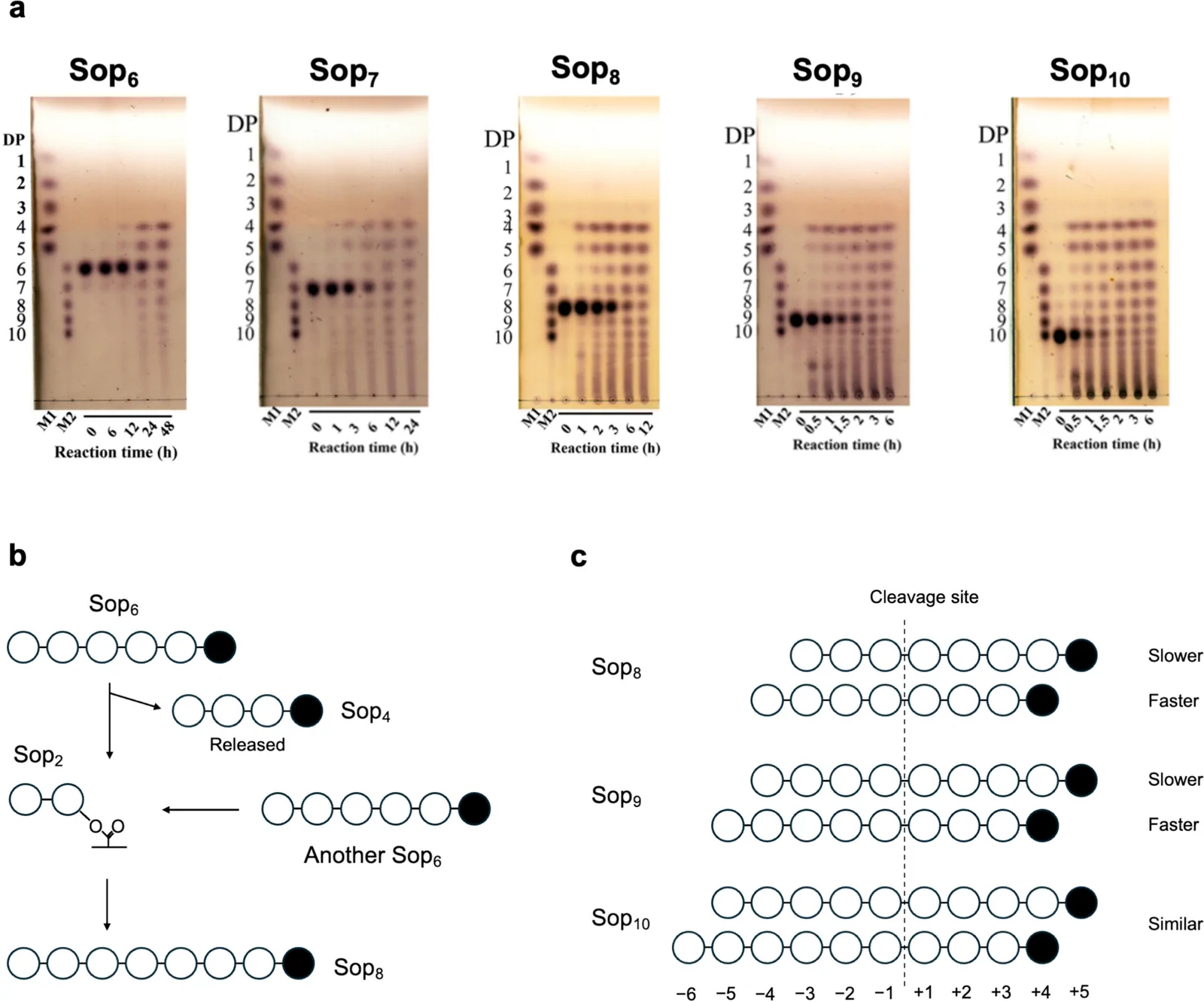
Chain length specificity of TiCGS_Tg_. **a** Action patterns towards Sop_6–10_ by TLC analysis. This result is cited from Tanaka et al. (2024). DPs shown at the left side of the TLC plate represent DPs of Sop_n_s. **b** Scheme of transglycosylation on Sop_6_. **c** Schematic representation of reaction patterns in the cases where Sop_4_ and Sop_5_ are released. Circles and lines represent Sop_n_s. Black circles represent glucose units at the reducing ends. Initial substrates (Sop_8–10_) in the solution are shown at the left side. Relative velocities of Sop_8–10_ production are shown on the right. Subsite numbers (plus numbers in the direction of reducing ends and minus towards non-reducing ends) are shown below the schematically depicted substrates

While a cyclic glucan is produced by intramolecular reaction, disproportionation occurs when two different substrate molecules participate in the reaction (Fig. 2). TiCGS_Tg_ disproportionates Sop_n_s with DP6 or higher but not with DP up to 5. When the enzyme is incubated with Sop_6_, only Sop_n_s with DP4 or higher are produced (Fig. 3a). In this case where Sop_4_ is released as a product, the substrate Sop_6_ is cleaved into Sop_4_ and Sop_2_ in the first step of the reaction (Fig. 3b). The Sop_4_ is released from the reducing end of the substrate, but Sop_2_ is bonded covalently with TiCGS_Tg_ in this step. If hydrolysis were to occur in the following step, Sop_2_ is also released as a product in addition to Sop_4_. However, Sop_2_ was not actually detected during the reaction. Instead, another Sop_6_ molecule acts on the intermediate to complete transglycosylation by producing Sop_8_. Overall, it is suggested that the enzyme catalyzes only transglycosylation without hydrolysis. It also indicates that at least four glucose units are necessary at the plus side of the subsite for the reaction to occur. In addition, when Sop_8–9_ are used as substrates, the amounts of Sop_4_, Sop_5_, and Sop_6_ produced are Sop_4_ > Sop_5_ > Sop_6_ at the initial stage of the reaction (Fig. 3c). It shows the tendency that a higher DP is preferable in the non-reducing end. Nevertheless, it is indifferent when a Sop with a higher DP, Sop_10_, is used as a substrate. In this case, Sop_4_ and Sop_5_ are produced at similar velocity. These results suggest that five glucose units at the minus side of the subsite are sufficient for high activity.

**Fig. 4:**
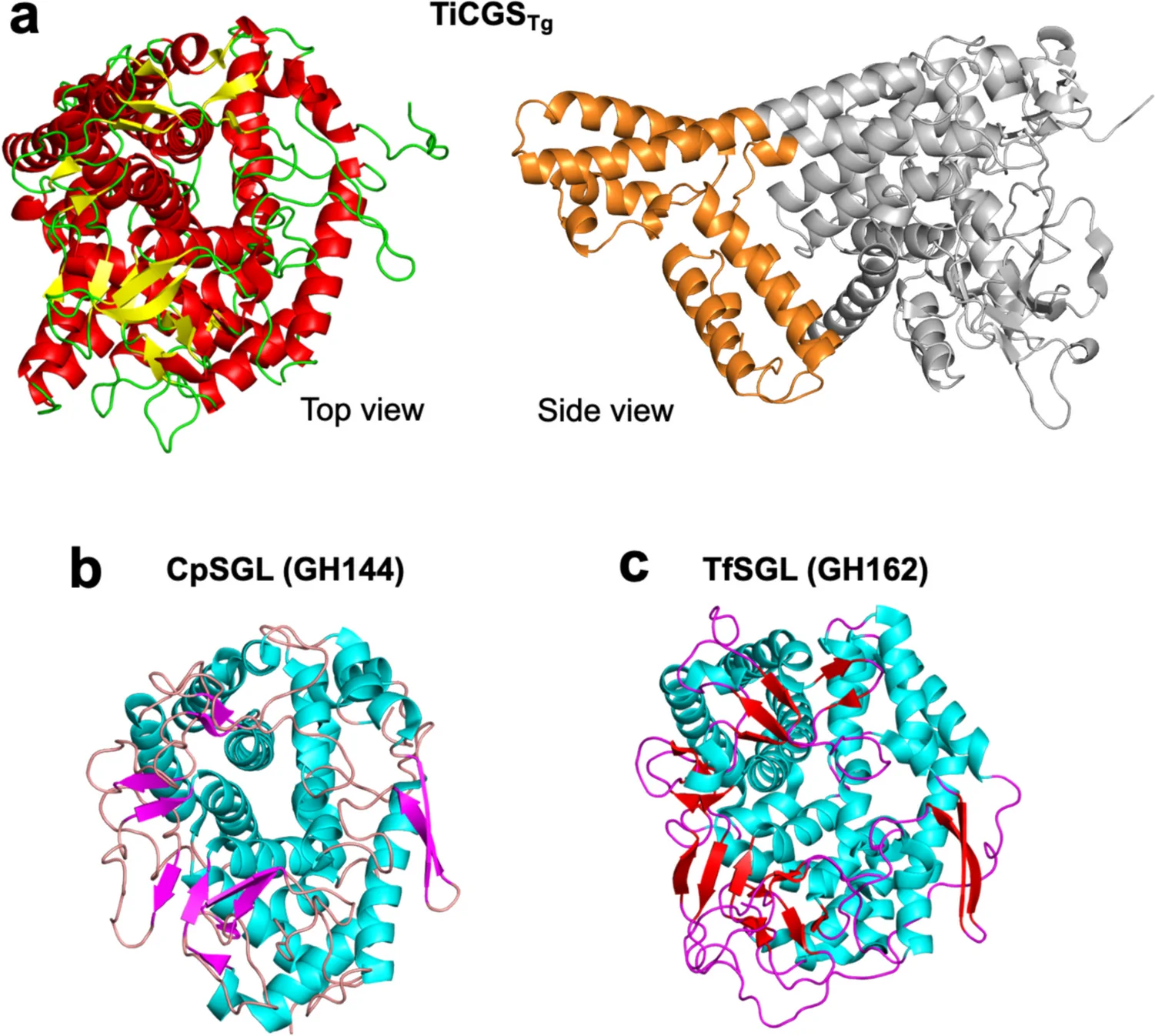
Overall structures of TiCGS_Tg_, CpSGL, and TfSGL. The structures (PDB ID: TiCGS_Tg_, 8WY1; CpSGL, 5GZK; TfSGL, 6IMW) are represented as cartoons. α-Helices and β-strands are shown in red and yellow (**a**, left), cyan and magenta (**b**), and cyan and orange (**c**), respectively. **a** (right) The insertion region that is not found in CpSGL nor TfSGL is shown in orange. The other region is shown in gray

**Fig. 5:**
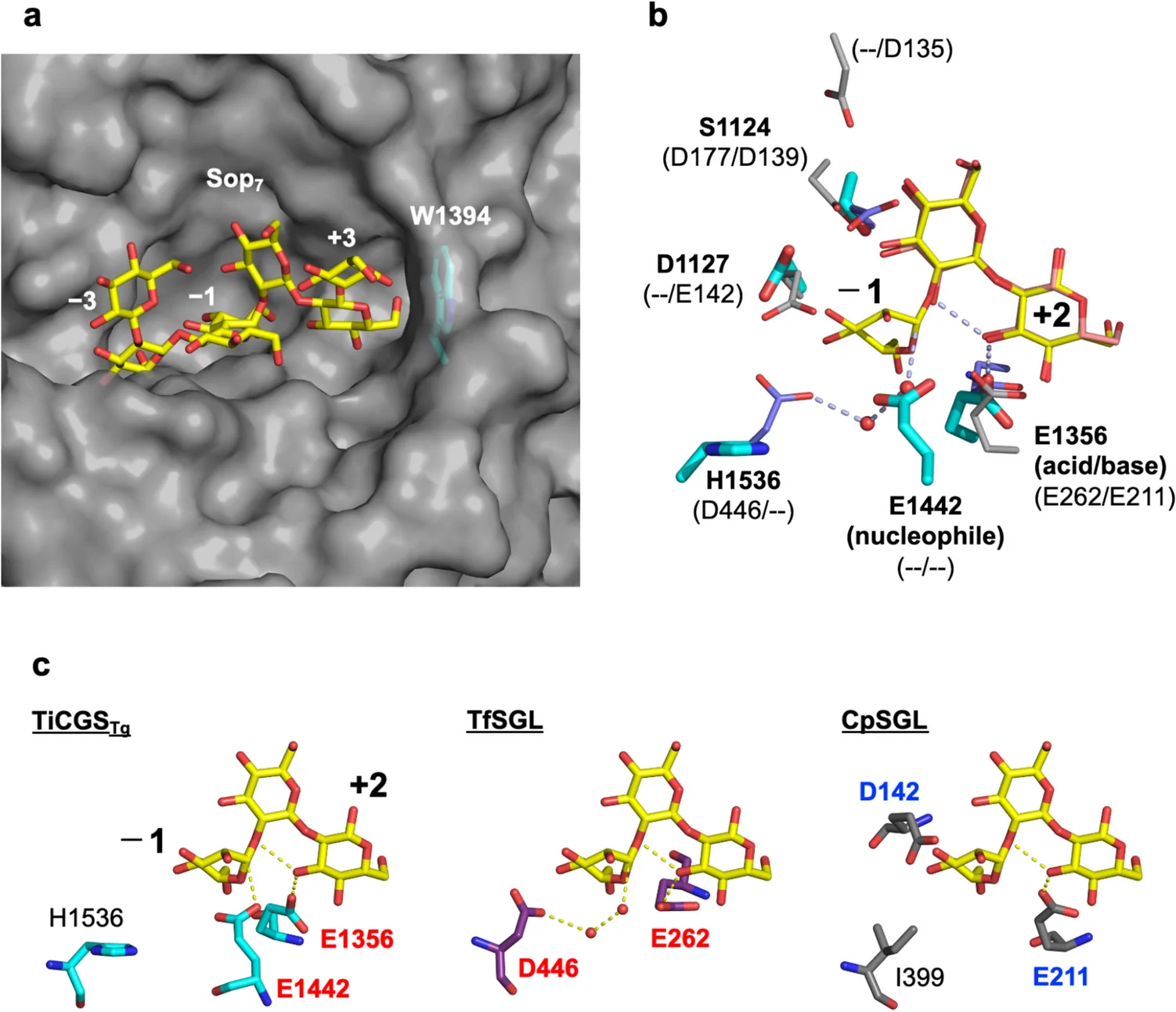
Catalytic site of TiCGS_Tg_. **a** Structure of TiCGS_Tg_ is represented as a gray surface. Sop_7_ in the Michaelis complex of TfSGL is shown as a yellow stick based on the superimposition of TiCGS_Tg_ (PDB ID, 8WY1) with TfSGL (E262Q mutant; PDB ID, 6IMW). Subsite numbers are shown in white letters. **b** Superimposition of catalytic residues and related proton-dissociable residues in TiCGS_Tg_, TfSGL (E262Q), and CpSGL. Residues in TiCGS_Tg_, TfSGL (E262Q), and CpSGL (PDB ID, 5GZK) are shown as cyan, light purple, and light gray sticks, respectively. Residue names of TiCGS_Tg_ are shown in bold letters with catalytic functions in parentheses. Residues in TfSGL (wild-type) and CpSGL corresponding to those in TiCGS_Tg_ are shown on the right and left respectively in parentheses). “--" symbols suggest that there are no corresponding residues. Ligands in the complex structures of TfSGL (E262Q) and CpSGL are shown as yellow and light pink sticks, respectively. Subsite numbers are shown in black letters. Only glucose units at subsites −1 to +2 in TfSGL are shown for visuality. **c** Comparisons of the reaction mechanism of TiCGS_Tg_ with TfSGL and CpSGL. Each catalytic site in **b** is displayed separately in **c** to compare reaction routes of the three enzymes. Reaction routes are shown as yellow dashed lines. Catalytic residues and candidates for catalytic residues are labeled with red and blue letters, respectively. Residues corresponding to D446 in TfSGL are labeled with black letters. A reaction route of nucleophilic attack has not yet been proposed for CpSGL

The overall ligand-free structure of TiCGS_Tg_ solved at 3.9 Å resolution is composed of a single (α/α)_6_-barrel fold (Tanaka et al. 2024) (Fig. 4a). The structure is similar to TfSGL (GH162) and CpSGL (GH144) although there is an insertion region composed of several α-helices (Fig. 4a right). When TiCGS_Tg_ and TfSGL-Sop_7_ are superimposed to reduce root mean square deviation with their main chains, the superimposed structure shows that the Sop_7_ molecule can be accommodated in the substrate pocket of TiCGS_Tg_ almost without steric hindrance (Fig. 5a). This observation is consistent with the activity of TiCGS_Tg_ on LβGs. Although the side chain of W1394 buries a space at subsite +4, there is still a space for the side chain of W1394 to flip into to allow potential space for the subsite.

In the superimposed structure, E1442 is located at a position where nucleophilic attack by this residue to an anomeric carbon atom at subsite −1 can occur (Fig. 5b). Indeed, E1442Q mutant did not show enzymatic activity on LβGs, indicating both structurally and functionally that E1442 is a nucleophile. E1356 corresponds to the general acid in TfSGL. Consistently, E1356Q showed remarkably reduced transglycosylation activity, suggesting that this residue is an acid/base catalyst. The reaction route that this residue donates a proton to the scissile bond oxygen atom in the first step of the enzymatic reaction is through the 3-OH group at subsite +2. This unique mechanism that the substrate OH group is utilized should be the reason why TiCGS_Tg_ does not show hydrolytic activity after formation of the enzyme–substrate intermediate (Fig. 2). Although a general base in CpSGL is not identified, the spatially conserved acidic amino acid between TiCGS_Tg_ and CpSGL (D1127 and E142, respectively) implies evolutional relationship between these two enzymes (Fig. 5b).

Although overall structures of TiCGS_Tg_, TfSGL (GH162), and CpSGL (GH144) are similar, their reaction mechanisms are different from each other due to the difference in the position of catalytic residues (or a candidate for a catalyst) (Fig. 5c). Thus, the phylogenetic group of TiCGS_Tg_ is classified into a newly created GH family, GH189 (Tanaka et al. 2024). GH families are further grouped into clan GHs, classification based on structural similarity. GH144, GH162, and GH189 families adopting single (α/α)_6_ folds have different positions of (potential) catalytic residues from other GH family enzymes with the same (α/α)_6_ folds. Based on these observations, a new clan (clan GH-S) for GH144 and GH162 was created. GH189 was regarded as a family related to clan GH-S, but it is not classified into this clan GH-S because this family follows an anomer-retaining mechanism unlike GH144 and GH162, which follow the anomer-inverting mechanism (B. Henrissat and N. Terrapon, personal communication). The definition of a new clan GH for GH189 awaits the discovery of another GH family which has a similar structure with the anomer-retaining mechanism.

### CGS from *R. radiobacter*

**Fig. 6:**
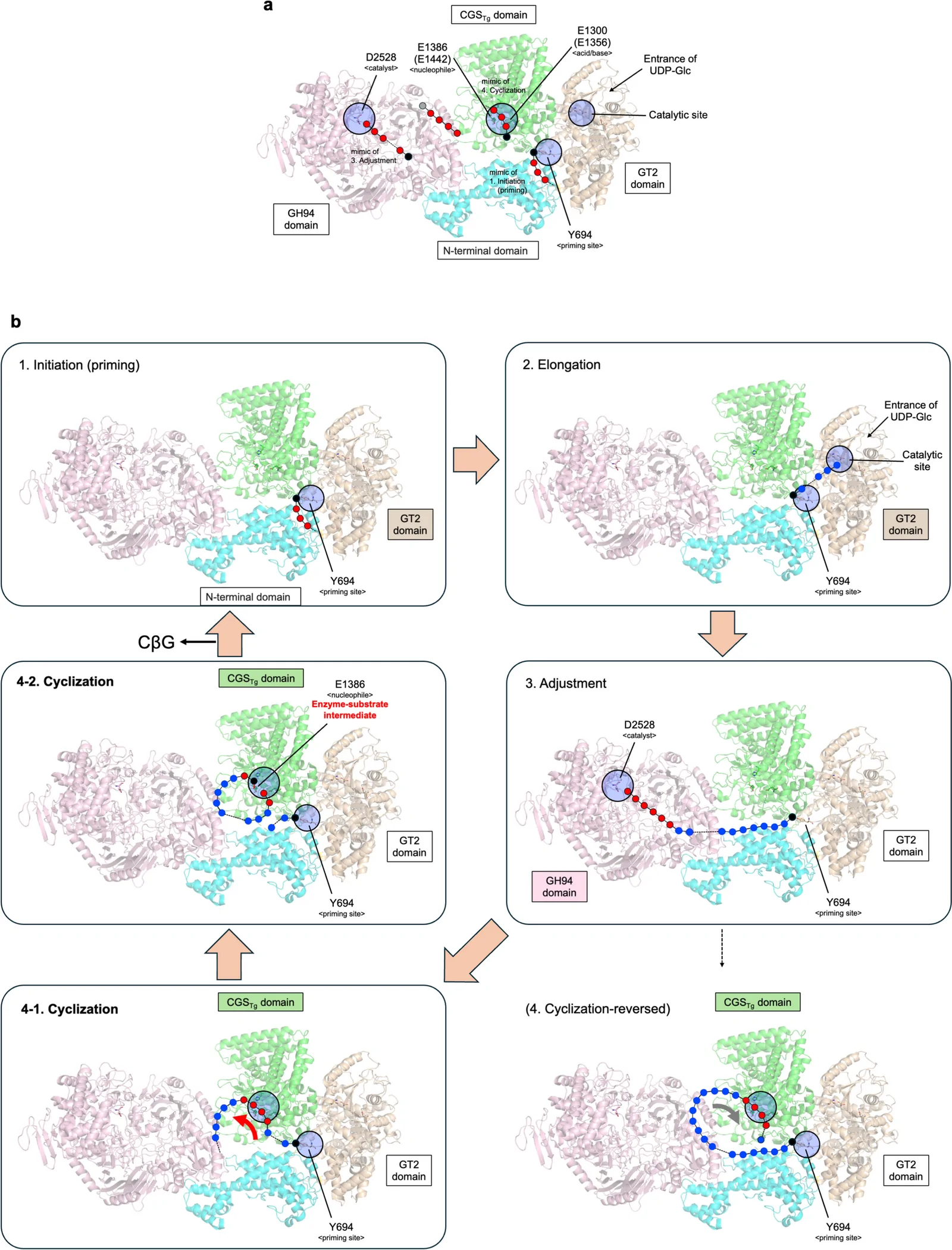
Perspectives on the reaction mechanism of CGS. The overall structure of RrCGS (PDB ID, 8RFE) is shown as a cartoon. The N-terminal domain, GT2 domain, CGS_Tg_ domain, and GH94 domain are shown in semi-transparent cyan, light brown, green, and light pink, respectively. The priming site (Y694) of a glucose unit in the GT2 domain, potential catalytic residues (E1300 and E1386) in the CGS_Tg_ domain, and catalytic residue (D2528) in GH94 domain are shown as sticks. These residues are highlighted with thin blue circles. Residues in parentheses (E1356 and E1442) are catalytic residues in TiCGS_Tg_ corresponding to E1300 and E1386 in RrCGS, respectively. **a** The Sop_n_s placed schematically where electron densities of ligands are observed. Red, gray, and black circles connected by solid and dashed lines are schematic representation of Sop_n_s. Gray and black both represent the reducing ends of the Sop_n_s but have different meanings. Black indicates the reducing end of the substrate whose direction is plausible judged from the enzymatic reactions, positions of catalytic residues and the priming site. Gray indicates that the plausibility of the direction cannot be judged either from these factors or known structures. Assigned states for Sop_n_s are labelled beside the Sop_n_s. The other fragment cannot be assigned to any state. **b** Schematic representation of the up-to-date reaction mechanism based on functional and structural analyses. Red circles represent Sop_n_ units which appear in **a**. The other parts of the substrates are complemented by blue circles. Substrate orientation indicated in (Sedzicki et al. 2024) in the cyclization step (bottom right panel). The red and gray arrows in the “cyclization” and “cyclization-reversed” panels, respectively, represent the conflicting directions of the substrate from the reducing end to the non-reducing end

After publication of functional and structural identification of TiCGS_Tg_, a study on overall structures of CGS from *R. radiobacter* C38 (RrCGS) using cryo-EM was published (Sedzicki et al. 2024) (Fig. 6a). By mass spectroscopy, the study on RrCGS successfully identified the tyrosine (Y694) as the residue in GT2 domain to which the first glucose molecule binds covalently in the priming process. The Y694A mutant lost ability to grow under osmotic stress condition, suggesting the importance of this residue. As this priming site is located in a large cavity accessible from cytoplasm, this cavity is regarded as a primer chamber (Fig. 6b top left). The overall direction of the fitted Sop_n_ molecule in the RrCGS structure is plausible in the sense that the reducing end of the Sop_n_ molecule is placed at the Y694 side. However, in the registered RrCGS structure, the electron density of the priming site is too unclear to fit a Sop_n_ molecule. Approximately half of the glucose units in the glucan chain fitted to the electron density are distorted. In addition, covalent bonds between a glucose unit and Y694 were not found in the structure. Therefore, elaborate electron density data is awaited to provide rigid structural evidence of the substrate in GT2 domain.

The catalytic site of GT2 domain involved in elongation of a primed Sop_n_ molecule is far from the priming site and is opposite to the GH94 domain (Fig. 6b top right). In reference to cellulose synthase from *C. sphaeroides* (BcsA) (Morgan et al. 2013), the direction of the substrate during elongation is plausible although, based on electron densities, detailed orientations of several ligands including UDP-glucose as a donor substrate are uncertain.

Electron densities that appear to be of substrates are also observed in the remaining CGS_Tg_ and GH94 domains of the RrCGS structure. The direction of the fitted Sop_n_ molecule in the GH94 domain is plausible based on the mechanism of adjustment step that the GH94 domain acts on the non-reducing end (Fig. 6a, b middle right). In contrast, the direction of the Sop_n_ molecule in the CGS_Tg_ domain cannot be judged due to its poor electron density nor can it be determined in the absence of a correct reaction mechanism in the CGS_Tg_ domain. Sedzicki et al. proposed an overall mechanism of CβG synthesis (Sedzicki et al. 2024). Nevertheless, in the scheme (Fig. 6 of Sedzicki et al. 2024), a β-1,2-glucan chain in CGS_Tg_ domain is assigned to be clockwise (when viewed from the same side as in Fig. 6b) in the reverse direction to those in the following registered structures: RrCGS, TfSGL (GH162), and TiCGS_Tg_ (the directions of the gray (clockwise) and red (counterclockwise) arrows in the bottom panels of Fig. 6b). We believe that cyclization cannot occur with the proposed clockwise direction (Fig. 6b bottom right) as the reaction mechanism of TiCGS_Tg_ (Fig. 2) evidenced in our report (Tanaka et al. 2024) indicated the counterclockwise direction (Fig. 6b bottom left). In view of the structural similarity between CGS_Tg_ and TfSGL, the β-1,2-glucan chain is expected to form the enzyme–substrate intermediate through binding to the CGS_Tg_ domains in the same orientation as in TfSGL (Fig. 6b middle left). The whole structure of TiCGS is required to understand the detailed reaction mechanism for CβG synthesis.

### Enzymes synthesizing CβGαs

**Fig. 7:**
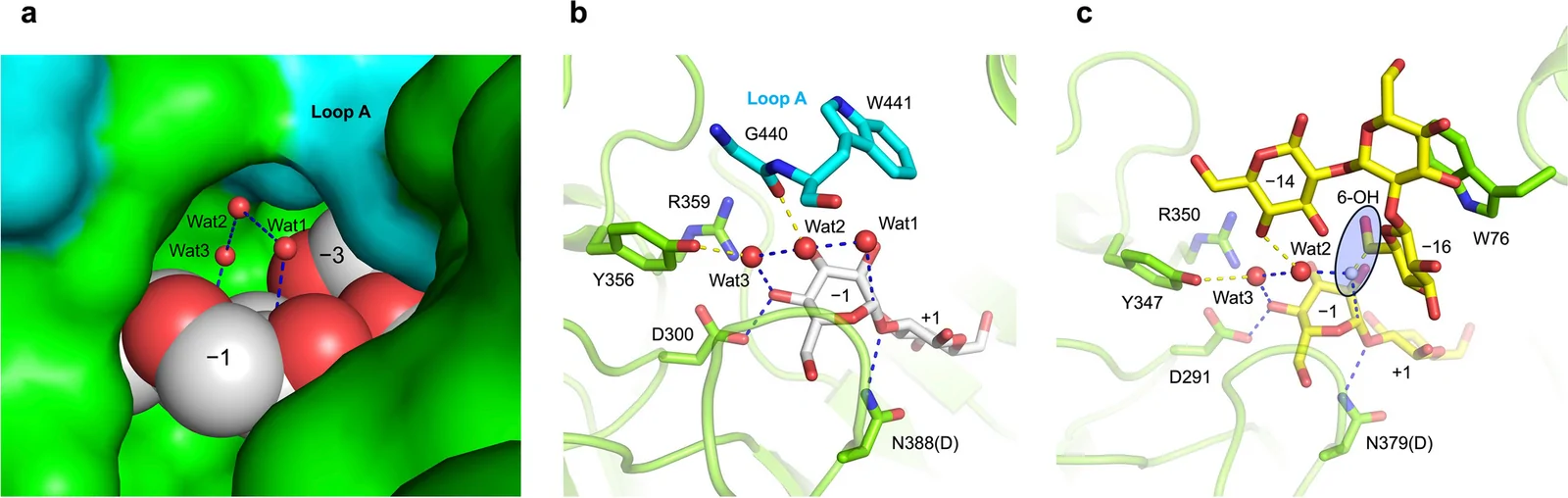
Catalytic sites of EcOpgD and XccOpgD. **a** Sequestration of water molecules from solvent in EcOpgD. The complex of EcOpgD (D388N mutant) with a β-1,2-glucan is shown (PDB ID, 8IP1). The two subunits in the dimer of EcOpgD are shown as green and cyan surfaces, respectively. The residues in Loop A of the cyan subunit are shown. Water molecules are in small red spheres. The water molecules are denoted as Wat1, Wat2, and Wat3 in the order of distance from the nucleophile. Glucose units at subsites −1 and −3 are shown as spheres. Blue dashed lines represent the reaction route. **b**, **c** Reaction routes of EcOpgD (**b**) and XccOpgD (**c**). The complexes of EcOpgD (D388N mutant) and XccOpgD (D379N mutant) with a β-1,2-glucan are shown (PDB IDs, 8IP1 and 8X18, respectively). Parentheses represent corresponding residues in the wild-type enzymes. The reaction routes are shown as blue dashed lines. Yellow dashed lines represent hydrogen bonds with the water molecules in the reaction routes. Color codes in (**b**) are the same as in **a**. **c** The substrate is shown as yellow sticks. The white small sphere represents Wat1 from EcOpgD superimposed to XccOpgD. The highlighted light blue circle suggests that the 6-OH group can be located at the position of the white small sphere by flipping the methylene group of the glucose unit at subsite −16

The discovery of CβGα synthase is led by the identification of OpgG and OpgD from *E. coli* involved in OPG synthesis as SGLs. OpgG and OpgD belong to an enzyme group that does not show any amino acid sequence homology to a superfamily including GH162, GH144, and GH189. Although a ligand-free structure of OpgG is available (Hanoulle et al. 2004), it does not decipher the biochemical function of OpgG. The finding of biochemical functions of OpgG and OpgD resulted in creation of a new GH family, GH186 (Motouchi et al. 2023). One of the most remarkable features of OpgD is that a loop, which is named Loop A, sequesters the proton relay route from the solvent (Fig. 7a, b). It is suggested that OpgD achieves efficient reaction velocity by this sequestration. Despite the indispensable feature of OpgD, the amino acid sequence in Loop A is conserved only in a limited range among GH186 homologs. Therefore, we expect that GH186 contains members having different reaction mechanisms.

**Fig. 8:**
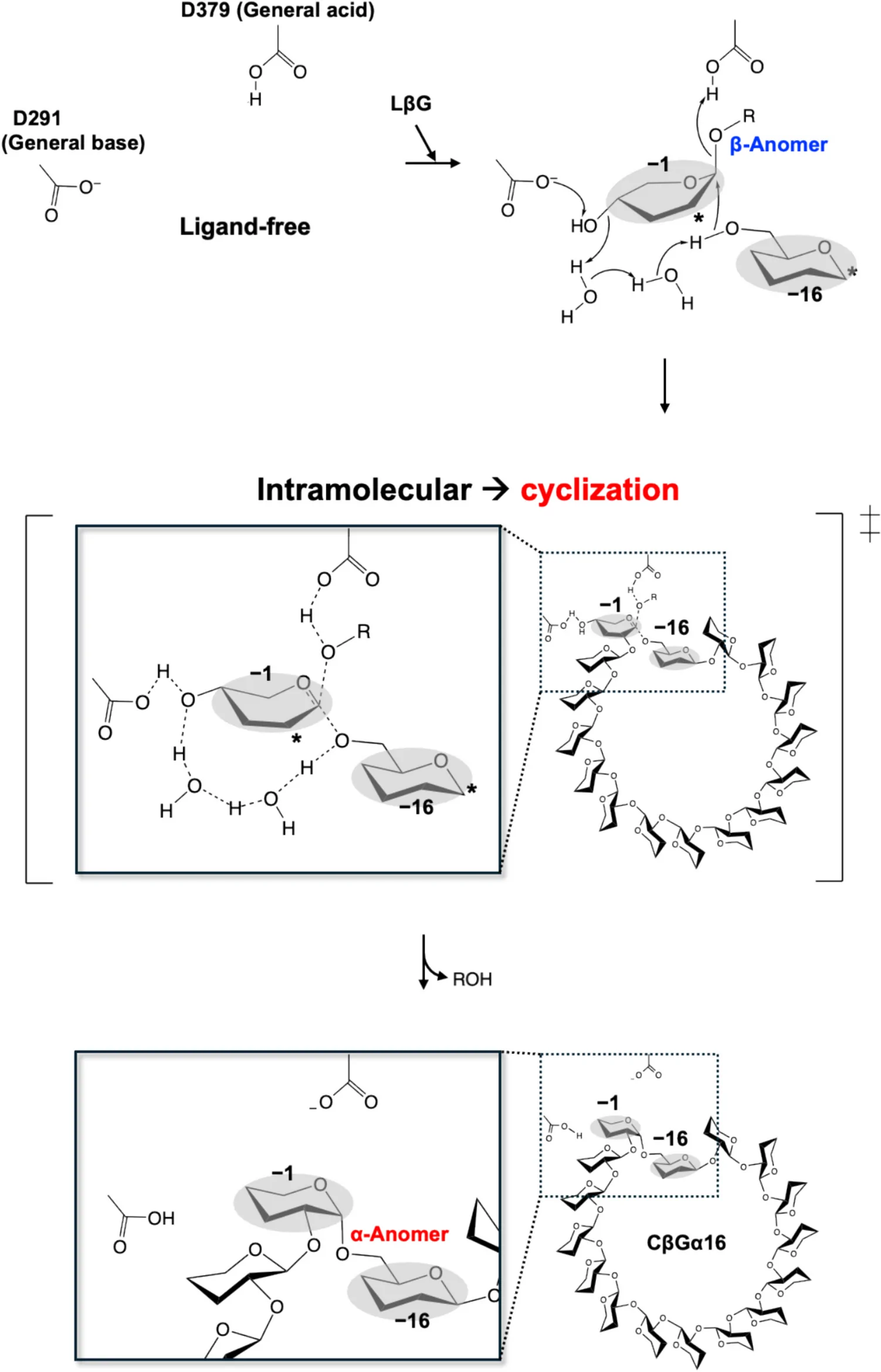
The reaction mechanism of anomer-inverting transglycosylation in XccOpgD. Enzyme–substrate intermediate is formed by direct protonation of a scissile bond oxygen atom by D379 (general acid). The 6-OH group in the glucose unit at subsite −16 is activated by D291 (general base) through two water molecules and the 4-OH group in the glucose unit at subsite −1. Nucleophilic attack by the activated 6-OH group occurs. This reaction occurs only intramolecularly. In the middle panel showing the intermediate, water molecules shown in the enlarged view on the left are omitted in the structure on the right. Finally, α-anomer is formed from β-anomer to cyclize a substrate LβG. Representation of carbohydrates is partially omitted for visuality. Asterisks indicate that they are linked with a Sop_14_ unit

*X. campestris* is a phytopathogen producing CβGα and possesses a gene encoding another GH186 homolog (XccOpgD). Although XccOpgD is a close homolog of EcOpgD, XccOpgD produces only CβGα with DP16 from substrate LβGs (Motouchi et al. 2024). The ESI–MS and NMR analyses of the products indicated that XccOpgD carries out anomer-inverting transglycosylation. In the Michaelis complex with LβG molecules, no nucleophilic water was observed. Instead, the 6-OH group of a glucose unit at subsite −16 was located in the vicinity of subsite −1 (Fig. 7b, c). The oxygen atom of the hydroxy group is located at the position in which a nucleophile should reside when the hydroxy group is flipped. This observation indicated that the reaction mechanism of anomer-inverting transglycosylation was clearly evidenced in terms of tertiary structure for the first time (Fig. 8).

## Remarks

This review mainly focused on functions and structures of key enzymes that catalyze cyclization of β-1,2-glucans. Cyclic β-1,2-glucans play key roles in physiologically important interactions between organisms such as symbiosis and pathogenesis. Despite the relevance of the glucans to these interactions, the unveiled variety of enzymes associated with the glucans is limited at present. Mining the genomes by further exploration of homologs of known GHs and gene clusters is desired. This review also introduced discoveries of the new reaction mechanisms in relation to recently found enzymes. Findings of new enzymes will be a basis for further understanding of the roles of these glucans and applications such as the development of pesticides for phytopathogens and provide new platforms and ideas for designing enzymes with new chemical reactions.

## Footnotes

1 “S” is used for abbreviations to distinguish linkage positions clearly from other glucans or enzymes acting on them. “S” is derived from “sophorooligosaccharide”, an alternative name of β-1,2-glucooligosaccharide.

## Abbreviations

CβG: Cyclic β-1,2-glucan
DP: Degree of polymerization
CβGα: α-1,6-Cyclized β-1,2-glucan
LβG: Linear β-1,2-glucan
OPG: Osmoregulated periplasmic glucans
Sop_n_: β-1,2-Glucooligosaccharide (n is DP)
CGS: Cyclic β-1,2-glucan synthase
SOGP: 1,2-β-Oligoglucan phosphorylase
SGL: β-1,2-Glucanase
CpSGL: SGL from *C. pinensis*
TfSGL: SGL from *T. funiculosus*
GH: Glycoside hydrolases
GT: Glycosyltransferase
TiCGS: CGS homolog from *T. italicus*
TiCGS_Tg_: The conserved domain in the middle region of TiCGS
RrCGS: CGS from *R. radiobacter* C38
XccOpgD: GH186 homolog from *X. campestris* pv. *campestris*

## References

[CR1] Abe K, Nakajima M, Kitaoka M, Toyoizumi H, Takahashi Y, Sugimoto N, Nakai H, Taguchi H (2015) Large-scale preparation of 1,2-β-glucan using 1,2-β-oligoglucan phosphorylase. J Appl Glycosci 62(2):47–52. 10.5458/jag.jag.JAG-2014_011

[CR2] Abe K, Nakajima M, Yamashita T, Matsunaga H, Kamisuki S, Nihira T, Takahashi Y, Sugimoto N, Miyanaga A, Nakai H, Arakawa T, Fushinobu S, Taguchi H (2017) Biochemical and structural analyses of a bacterial *endo*-β-1,2-glucanase reveal a new glycoside hydrolase family. J Biol Chem 292(18):7487–7506. 10.1074/jbc.M116.762724 28270506 10.1074/jbc.M116.762724 PMC5418048

[CR3] Ahmad D, Ying Y, Bao J (2024) Understanding starch biosynthesis in potatoes for metabolic engineering to improve starch quality: a detailed review. Carbohydr Polym 346:122592. 10.1016/j.carbpol.2024.122592 39245484 10.1016/j.carbpol.2024.122592

[CR4] Amemura A, Hashimoto T, Koizumi K, Utamura T (1985) Occurrence of extracellular (1→2)-β-d-glucans and (1→2)-β-d-gluco-oligosaccharides in *Acetobacter*. J Gen Microbiol 131:301–307

[CR5] Arellano-Reynoso B, Lapaque N, Salcedo S, Briones G, Ciocchini AE, Ugalde R, Moreno E, Moriyón I, Gorvel JP (2005) Cyclic β-1,2-glucan is a brucella virulence factor required for intracellular survival. Nat Immunol 6:618–625. 10.1038/ni1202 15880113 10.1038/ni1202

[CR6] Bhagwat AA, Jun W, Liu L, Kannan P, Dharne M, Pheh B, Tall BD, Kothary MH, Gross KC, Angle S, Meng J, Smith A (2009) Osmoregulated periplasmic glucans of *Salmonella enterica* serovar Typhimurium are required for optimal virulence in mice. Microbiology (Reading) 155(1):229–237. 10.1099/mic.0.023747-0 19118363 10.1099/mic.0.023747-0

[CR7] Bohin J (1996) Cell-associated glucans of *Burkholderia solanacearum* and *Xanthomonas campestris* pv. *citri*: a new family of periplasmic glucans. J Bacteriol 178(8):2263–2271. 10.1128/jb.178.8.2263-2271.1996 10.1128/jb.178.8.2263-2271.1996 PMC177934 8636027

[CR8] Bontemps-Gallo S, Bohin JP, Lacroix JM (2017) Osmoregulated Periplasmic Glucans. Ecosal Plus 7(2):1–17. 10.1128/ecosalplus.esp-0001-2017 10.1128/ecosalplus.esp-0001-2017 PMC11575687 28593831

[CR9] Briones G, Iñón de Iannino N, Steinberg M, Ugalde RA (1997) Periplasmic cyclic 1,2-β-glucan in *Brucella* spp. is not osmoregulated. Microbiology (N Y) 143(4):1115–1124. 10.1099/00221287-143-4-1115 10.1099/00221287-143-4-1115 9141674

[CR10] Castro OA, Zorreguieta A, Ielmini V, Vega G, Ielpi L (1996) Cyclic β-(1,2)-glucan synthesis in *Rhizobiaceae*: roles of the 319-kilodalton protein intermediate. J Bacteriol 178(20):6043–6048. 10.1128/jb.178.20.6043-6048.1996 8830704 10.1128/jb.178.20.6043-6048.1996 PMC178464

[CR11] Chen Y, Zhang KX, Liu H, Zhu Y, Bu QY, Song SX, Li YC, Zou H, You XY, Zhao GP (2024) Impact of ginsenoside Rb1 on gut microbiome and associated changes in pharmacokinetics in rats. Sci Rep 14(1):21168. 10.1038/s41598-024-72225-1 39256599 10.1038/s41598-024-72225-1 PMC11387729

[CR12] Ciocchini AE, Roset MS, Briones G, Iñón de Iannino N, Ugalde RA (2006) Identification of active site residues of the inverting glycosyltransferase Cgs required for the synthesis of cyclic β-1,2-glucan, a *Brucella abortus* virulence factor. Glycobiology 16(7):679–691. 10.1093/glycob/cwj113 16603625 10.1093/glycob/cwj113

[CR13] Ciocchini AE, Guidolin LS, Casabuono AC, Couto AS, de Iannino NI, Ugalde RA (2007) A glycosyltransferase with a length-controlling activity as a mechanism to regulate the size of polysaccharides. Proc Natl Acad Sci U S A 104(42):16492–16497. 10.1073/pnas.0708025104 17921247 10.1073/pnas.0708025104 PMC2034269

[CR14] Cochard C, Caby M, Gruau P, Madec E, Marceau M, Macavei I, Lemoine J, Le Danvic C, Bouchart F, Delrue B, Bontemps-Gallo S, Lacroix JM (2023) Emergence of the *Dickeya* genus involved duplication of the OmpF porin and the adaptation of the EnvZ-OmpR signaling network. Microbiol Spectr 11(5):e0083323. 10.1128/spectrum.00833-23 37642428 10.1128/spectrum.00833-23 PMC10581057

[CR15] Cosgrove DJ, Dupree P, Gomez ED, Haigler CH, Kubicki JD, Zimmer J (2024) How many glucan chains form plant cellulose microfibrils? A Mini Review Biomacromolecules 25(10):6357–6366. 10.1021/acs.biomac.4c00995 39207939 10.1021/acs.biomac.4c00995 PMC11480985

[CR16] Daitch AK, Smith EL, Goley ED (2024) OpgH is an essential regulator of *Caulobacter* morphology. mBio 15(9):e0144324. 10.1128/mbio.01443-24 39145657 10.1128/mbio.01443-24 PMC11389396

[CR17] Drula E, Garron ML, Dogan S, Lombard V, Henrissat B, Terrapon N (2022) The carbohydrate-active enzyme database: functions and literature. Nucleic Acids Res 50(1):571–577. 10.1093/nar/gkab1045 10.1093/nar/gkab1045 PMC8728194 34850161

[CR18] Dylan T, Helinski DR, Ditta GS (1990) Hypoosmotic adaptation in *Rhizobium meliloti* requires β-(1→2)-glucan. J Bacteriol 172(3):1400–1408. 10.1128/jb.172.3.1400-1408.1990 1689716 10.1128/jb.172.3.1400-1408.1990 PMC208612

[CR19] Frusciante L, Geminiani M, Shabab B, Olmastroni T, Scavello G, Rossi M, Mastroeni P, Nyong’a CN, Salvini L, Lamponi S, Parisi ML, Sinicropi A, Costa L, Spiga O, Trezza A, Santucci A (2024) Exploring the antioxidant and anti-inflammatory potential of Saffron (*Crocus sativus*) tepals extract within the circular bioeconomy. Antioxidants 13(9):1082. 10.3390/antiox13091082 39334741 10.3390/antiox13091082 PMC11428576

[CR20] Fuertes-Rabanal M, Largo-Gosens A, Fischer A, Munzert KS, Carrasco-López C, Sánchez-Vallet A, Engelsdorf T, Mélida H (2024) Linear β-1,2-glucans trigger immune hallmarks and enhance disease resistance in plants. J Exp Bot 75(22):7337–7350. 10.1093/jxb/erae368 39225413 10.1093/jxb/erae368 PMC11630039

[CR21] Gay-Fraret J, Ardissone S, Kambara K, Broughton WJ, Deakin WJ, Le Quéré A (2012) Cyclic-β-glucans of *Rhizobium* (*Sinorhizobium*) sp. strain NGR234 are required for hypo-osmotic adaptation, motility, and efficient symbiosis with host plants. FEMS Microbiol Lett 333(1):28–36. 10.1111/j.1574-6968.2012.02595.x 10.1111/j.1574-6968.2012.02595.x 22583376

[CR22] Guidolin LS, Morrone Seijo SM, Guaimas FF, Comerci DJ, Ciocchinia AE (2015) Interaction network and localization of *Brucella abortus* membrane proteins involved in the synthesis, transport, and succinylation of cyclic β-1,2-glucans. J Bacteriol 197(9):1640–1648. 10.1128/JB.00068-15 25733613 10.1128/JB.00068-15 PMC4403662

[CR23] Hanoulle X, Rollet E, Clantin B, Landrieu I, Ödberg-Ferragut C, Lippens G, Bohin JP, Villeret V (2004) Structural analysis of *Escherichia coli* OpgG, a protein required for the biosynthesis of osmoregulated periplasmic glucans. J Mol Biol 342(1):195–205. 10.1016/j.jmb.2004.07.004 15313617 10.1016/j.jmb.2004.07.004

[CR24] Hisamatsu M, Amemura A, Matsuo T, Matsuda H, Harada T (1982) Cyclic (1→2)-β-d-glucan and the octasaccharide repeating-unit of succinoglycan produced by *Agrobactenium*. J Gen Microbiol 128:1873–1879

[CR25] Iannino N, Briones G, Tolmasky M, Ugalde R (1998) Molecular cloning and characterization of *cgs*, the *Brucella abortus* cyclic β(1–2) glucan synthetase gene: genetic complementation of *Rhizobium meliloti ndvB* and *Agrobacterium tumefaciens chvB* mutants. J Bacteriol 180(17):4392–4400. 10.1128/JB.180.17.4392-4400.1998 9721274 10.1128/jb.180.17.4392-4400.1998 PMC107446

[CR26] Javvadi S, Pandey SS, Mishra A, Pradhan BB, Chatterjee S (2018) Bacterial cyclic β-(1,2)-glucans sequester iron to protect against iron-induced toxicity. EMBO Rep 19(1):172–186. 10.15252/embr.201744650 29222343 10.15252/embr.201744650 PMC5757255

[CR27] Kennedy EP (1982) Osmotic regulation and the biosynthesis of membrane derived oligosaccharides in *Escherichia coli*. Proc Natl Acad Sci U S A 79(4):1092–1095. 10.1073/pnas.79.4.1092 7041113 10.1073/pnas.79.4.1092 PMC345906

[CR28] Kim TH, Ku SK, Lee IC, Bae JS (2012) Anti-inflammatory effects of kaempferol-3-*O*-sophoroside in human endothelial cells. Inflamm Res 61(3):217–224. 10.1007/s00011-011-0403-9 22113342 10.1007/s00011-011-0403-9

[CR29] Kobayashi K, Shimizu H, Tanaka N, Kuramochi K, Nakai H, Nakajima M, Taguchi H (2022) Characterization and structural analyses of a novel glycosyltransferase acting on the β-1,2-glucosidic linkages. J Biol Chem 298(3):101606. 10.1016/j.jbc.2022.101606 35065074 10.1016/j.jbc.2022.101606 PMC8861115

[CR30] Lequette Y, Ödberg-Ferragut C, Bohin JP, Lacroix JM (2004) Identification of *mdoD*, an *mdoG* paralog which encodes a twin-arginine-dependent periplasmic protein that controls osmoregulated periplasmic glucan backbone structures. J Bacteriol 186(12):3695–3702. 10.1128/JB.186.12.3695-3702.2004 15175282 10.1128/JB.186.12.3695-3702.2004 PMC419940

[CR31] Mahasenan KV, Batuecas MT, De Benedetti S, Kim C, Rana N, Lee M, Hesek D, Fisher JF, Sanz-Aparicio J, Hermoso JA, Mobashery S (2020) Catalytic cycle of glycoside hydrolase BglX from *Pseudomonas aeruginosa* and its implications for biofilm formation. ACS Chem Biol 15(1):189–196. 10.1021/acschembio.9b00754 31877028 10.1021/acschembio.9b00754 PMC7995829

[CR32] Managa MG, Sultanbawa Y, Sivakumar D (2020) Effects of different drying methods on untargeted phenolic metabolites, and antioxidant activity in Chinese cabbage (*Brassica rapa* L. subsp. chinensis) and Nightshade (*Solanum retroflexum* Dun.). Molecules 25(6):1326. 10.3390/molecules25061326 10.3390/molecules25061326 PMC7145292 32183223

[CR33] McIntire FC, Peterson WH, Riker AJ (1940) Factors influencing the carbon metabolism of the crown gall organism. J Agric Res 61(4):313–320

[CR34] Meng J, Xu J, Chen J (2020) The role of osmoregulated periplasmic glucans in the biofilm antibiotic resistance of *Yersinia enterocolitica*. Microb Pathog 147:104284. 10.1016/j.micpath.2020.104284 32492459 10.1016/j.micpath.2020.104284

[CR35] Mohamed GA, El-Agamy DS, Abdallah HM, Sindi IA, Almogaddam MA, Alzain AA, Andijani YS, Ibrahim SRM (2024) Kaempferol sophoroside glucoside mitigates acetaminophen-induced hepatotoxicity: role of Nrf2/NF-κB and JNK/ASK-1 signaling pathways. Heliyon 10810:e31448. 10.1016/j.heliyon.2024.e31448 10.1016/j.heliyon.2024.e31448 PMC11133934 38813141

[CR36] Molina A, Jordá L, Torres MÁ, Martín-Dacal M, Berlanga DJ, Fernández-Calvo P, Gómez-Rubio E, Martín-Santamaría S (2024) Plant cell wall-mediated disease resistance: current understanding and future perspectives. Mol Plant 17(5):699–724. 10.1016/j.molp.2024.04.003 38594902 10.1016/j.molp.2024.04.003

[CR37] Molina A, Sánchez-Vallet A, Jordá L, Carrasco-López C, Rodríguez-Herva JJ, López-Solanilla E (2024) Plant cell walls: source of carbohydrate-based signals in plant-pathogen interactions. Curr Opin Plant Biol 82:102630. 10.1016/j.pbi.2024.102630 39306957 10.1016/j.pbi.2024.102630

[CR38] Morgan JLW, Strumillo J, Zimmer J (2013) Crystallographic snapshot of cellulose synthesis and membrane translocation. Nature 493(7431):181–186. 10.1038/nature11744 23222542 10.1038/nature11744 PMC3542415

[CR39] Motouchi S, Kobayashi K, Nakai H, Nakajima M (2023) Identification of enzymatic functions of osmo-regulated periplasmic glucan biosynthesis proteins from *Escherichia coli* reveals a novel glycoside hydrolase family. Commun Biol 6:961. 10.1038/s42003-023-05336-6 37735577 10.1038/s42003-023-05336-6 PMC10514313

[CR40] Motouchi S, Komba S, Nakai H, Nakajima M (2024) Discovery of anomer-inverting transglycosylase: cyclic glucohexadecaose-producing enzyme from *Xanthomonas*, a phytopathogen. J Am Chem Soc 146(26):17738–17746. 10.1021/jacs.4c02579 38957137 10.1021/jacs.4c02579 PMC11228985

[CR41] Murakami K, Nasu H, Fujiwara T, Takatsu N, Yoshida N, Furuta K, Kaito C (2021) The absence of osmoregulated periplasmic glucan confers antimicrobial resistance and increases virulence in *Escherichia coli*. J Bacteriol 203(12):e00515-e520. 10.1128/JB.00515-20 33846116 10.1128/JB.00515-20 PMC8316038

[CR42] Naim N, Bouymajane A, Oulad El Majdoub Y, Ezrari S, Lahlali R, Tahiri A, Ennahli S, Laganà Vinci R, Cacciola F, Mondello L, Madani I (2022) Flavonoid composition and antibacterial properties of *Crocus sativus* L. petal extracts. Molecules 28(1):186. 10.3390/molecules28010186 10.3390/molecules28010186 PMC9822159 36615378

[CR43] Nakajima M (2023) β-1,2-Glucans and associated enzymes. Biologia (Bratisl) 78(7):1741–1757. 10.1007/s11756-022-01205-5

[CR44] Nakajima M, Toyoizumi H, Abe K, Nakai H, Taguchi H, Kitaoka M (2014) 1,2-β-oligoglucan phosphorylase from *Listeria innocua*. PLoS ONE 9(3):e92353. 10.1371/journal.pone.0092353 24647662 10.1371/journal.pone.0092353 PMC3960220

[CR45] Nakajima M, Tanaka N, Furukawa N, Nihira T, Kodutsumi Y, Takahashi Y, Sugimoto N, Miyanaga A, Fushinobu S, Taguchi H, Nakai H (2017) Mechanistic insight into the substrate specificity of 1,2-β-oligoglucan phosphorylase from *Lachnoclostridium phytofermentans*. Sci Rep 7:42671. 10.1038/srep42671 28198470 10.1038/srep42671 PMC5309861

[CR46] Nakajima M, Tanaka N, Kobayashi K, Nakai H, Kimura S, Iwata T, Taguchi H (2021) Enzymatic control and evaluation of degrees of polymerization of β-(1→2)-glucans. Anal Biochem 632:114366. 10.1016/j.ab.2021.114366 34509443 10.1016/j.ab.2021.114366

[CR47] Qu X, Ji Y, Long J, Zheng D, Qiao Z, Lin Y, Lu C, Zhou Y, Cheng H (2025) Immuno- and gut microbiota-modulatory activities of β-1,6-glucans from *Lentinus edodes*. Food Chem 466:142209. 10.1016/j.foodchem.2024.142209 39612846 10.1016/j.foodchem.2024.142209

[CR48] Rigano LA, Payette C, Brouillard G, Marano MR, Abramowicz L, Torres PS, Yun M, Castagnaro AP, Oirdi ME, Dufour V, Malamud F, Dow JM, Bouarab K, Vojnov AA (2007) Bacterial cyclic β-(1,2)-glucan acts in systemic suppression of plant immune responses. Plant Cell 19(6):2077–2089. 10.1105/tpc.106.047944 17601826 10.1105/tpc.106.047944 PMC1955710

[CR49] Roset MS, Ciocchini AE, Ugalde RA, Iñón de Iannino N (2006) The *Brucella abortus* cyclic β-1,2-glucan virulence factor is substituted with *O*-ester-linked succinyl residues. J Bacteriol 188(14):5003–5013. 10.1128/JB.00086-06 16816173 10.1128/JB.00086-06 PMC1539967

[CR50] Sedzicki J, Ni D, Lehmann F, Wu N, Zenobi R, Jung S, Stahlberg H, Dehio C (2022) Mechanism of cyclic β-glucan export by ABC transporter Cgt of *Brucella*. Nat Struct Mol Biol 29(12):1170–1177. 10.1038/s41594-022-00868-7 36456825 10.1038/s41594-022-00868-7

[CR51] Sedzicki J, Ni D, Lehmann F, Stahlberg H, Dehio C (2024) Structure-function analysis of the cyclic β-1,2-glucan synthase from *Agrobacterium tumefaciens*. Nat Commun 15(1):1844. 10.1038/s41467-024-45415-8 38418509 10.1038/s41467-024-45415-8 PMC10901819

[CR52] Stone BA (2009) Chemistry of β-Glucans. Bacic A, Fincher BG, Stone BA(ed) Chemistry, biochemistry, and biology of 1–3 beta glucans and related polysaccharides. Elsevier, Amsterdam, pp 5–46

[CR53] Suárez ER, Bugden SM, Kai FB, Kralovec JA, Noseda MD, Barrow CJ, Grindley TB (2008) First isolation and structural determination of cyclic β-(1→2)-glucans from an alga, *Chlorella pyrenoidosa*. Carbohydr Res 343(15):2623–2633. 10.1016/j.carres.2008.07.009 18718577 10.1016/j.carres.2008.07.009

[CR54] Talaga P, Fournet B, Bohin JP (1994) Periplasmic glucans of *Pseudomonas syringae* pv. *syringae*. J Bacteriol 176(21):6538–6544. 10.1128/jb.176.21.6538-6544.1994 10.1128/jb.176.21.6538-6544.1994 PMC197007 7961404

[CR55] Talaga P, Cogez V, Wieruszeski JM, Stahl B, Lemoine J, Lippens G, Bohin JP (2002) Osmoregulated periplasmic glucans of the free-living photosynthetic bacterium *Rhodobacter sphaeroides*. Eur J Biochem 269(10):2464–2472. 10.1046/j.1432-1033.2002.02906.x 12027884 10.1046/j.1432-1033.2002.02906.x

[CR56] Tanaka N, Nakajima M, Narukawa-Nara M, Matsunaga H, Kamisuki S, Aramasa H, Takahashi Y, Sugimoto N, Abe K, Terada T, Miyanaga A, Yamashita T, Sugawara F, Kamakura T, Komba S, Nakai H, Taguchi H (2019) Identification, characterization, and structural analyses of a fungal *endo*-β-1,2-glucanase reveal a new glycoside hydrolase family. J Biol Chem 294(19):7942–7965. 10.1074/jbc.RA118.007087 30926603 10.1074/jbc.RA118.007087 PMC6514616

[CR57] Tanaka N, Saito R, Kobayashi K, Nakai H, Kamo S, Kuramochi K, Taguchi H, Nakajima M, Masaike T (2024) Functional and structural analysis of a cyclization domain in a cyclic β-1,2-glucan synthase. Appl Microbiol Biotechnol 108(1):187. 10.1007/s00253-024-13013-9 38300345 10.1007/s00253-024-13013-9 PMC10834661

[CR58] Wang Y, Berhow MA, Black M, Jeffery EH (2020) A comparison of the absorption and metabolism of the major quercetin in brassica, quercetin-3-*O*-sophoroside, to that of quercetin aglycone, in rats. Food Chem 311:125880. 10.1016/j.foodchem.2019.125880 31771913 10.1016/j.foodchem.2019.125880

[CR59] Wanke A, Malisic M, Wawra S, Zuccaro A (2021) Unraveling the sugar code: the role of microbial extracellular glycans in plant-microbe interactions. J Exp Bot 72(1):15–35. 10.1093/jxb/eraa414 32929496 10.1093/jxb/eraa414 PMC7816849

[CR60] Wieruszeski JM, Bohin A, Bohin JP, Lippens G (2001) In vivo detection of the cyclic osmoregulated periplasmic glucan of *Ralstonia solanacearum* by high-resolution magic angle spinning NMR. J Magn Reson 151(1):118–123. 10.1006/jmre.2001.2348 11444945 10.1006/jmre.2001.2348

[CR61] Wu J, Huang M, Liu H, Wu Y, Hu X, Wang J, Wang X (2024) Engineering *Escherichia coli* to efficiently produce colanic acid with low molecular mass and viscosity. J Agric Food Chem 72(28):15811–15822. 10.1021/acs.jafc.4c03187 38975865 10.1021/acs.jafc.4c03187

[CR62] Zhang P, Li Q, Chen Y, Peng N, Liu W, Wang X, Li Y (2022) Induction of cellulase production in *Trichoderma reesei* by a glucose-sophorose mixture as an inducer prepared using stevioside. RSC Adv 12(27):17392–17400. 10.1039/d2ra01192a 35765440 10.1039/d2ra01192a PMC9190947

[CR63] Zhang Y, Gong Y, Hu J, Zhang L, Benito MJ, Usmanov D, Nishanbaev SZ, Song X, Zou L, Wu Y (2025) Quercetin and kaempferol from saffron petals alleviated hydrogen peroxide-induced oxidative damage in B16 cells. J Sci Food Agric 105(2):967–973. 10.1002/jsfa.13887 39287449 10.1002/jsfa.13887

[CR64] Żurek N, Pycia K, Pawłowska A, Kapusta IT (2022) Phytochemical screening and bioactive properties of Juglans regia L. pollen. Antioxidants (Basel) 11(10):2046. 10.3390/antiox11102046 36290769 10.3390/antiox11102046 PMC9598064

